# Pathophysiological implication of reversed CT halo sign in invasive pulmonary mucormycosis: a rare case report

**DOI:** 10.1186/1746-1596-8-82

**Published:** 2013-05-17

**Authors:** Yoichiro Okubo, Takao Ishiwatari, Haruka Izumi, Fumitomo Sato, Kyoko Aki, Daisuke Sasai, Tsunehiro Ando, Minoru Shinozaki, Kazuhiko Natori, Naobumi Tochigi, Megumi Wakayama, Yoshinobu Hata, Haruo Nakayama, Tetsuo Nemoto, Kazutoshi Shibuya

**Affiliations:** 1 Department of Surgical Pathology, Toho University School of Medicine, 6-11-1 Omori-Nishi, Ota-Ku, Tokyo 143-8541, Japan; 2 Division of Hematology and Oncology, Department of Medicine, Toho University Medical Center, 6-11-1 Omori-Nishi, Ota-Ku, Tokyo 143-8541, Japan; 3 Department of Chest Surgery, Toho University Medical Center, 6-11-1 Omori-Nishi, Ota-Ku, Tokyo 143-8541, Japan; 4 Department of Respiratory Medicine, Japanese Red Cross Medical Center, 4-1-22, Hiroo Shibuya-Ku, Tokyo 150-8935, Japan; 5 Department of Neurosurgery, Toho University Ohashi Medical Center, 2-17-6, Ohashi, Meguro, Tokyo 153-8515, Japan; 6 Department of Dermatology, Peking University First Hospital, Beijing, China

**Keywords:** *Reversed halo sign*, *Mucormycosis*, *Aspergillosis*, *Discrete nodule*

## Abstract

**Background:**

It has been accepted that reversed halo sign (RHS) appeared on a computed tomography (CT) image in immunocompromised patients indicates an invasive fungal infection, but its pathophysiology remains obscure as to what this image implies. Therefore, the present report describes detailed radiological and histopathological findings of a case of invasive pulmonary mucormycosis (IPM) presenting RHS with comparison to those from a lesion of discrete nodule caused by invasive pulmonary aspergillosis (IPA), and discusses the pathophysiological implications of this characteristic image.

**Case presentation:**

RHS had been clinically noted at the time of recovering of bone marrow function of a 64-year-old Japanese man who had chemotherapy for his acute lymphoblastic leukemia. Histological examination of the surgically removed lung revealed a lesion of IPM. This was composed of coagulation necrosis of septa at the center of lesion with preservation of air content which was encompassed outer rim comprising triplet structure; liquefaction, consolidation, and organization from the inner to the outer layer. In addition, Micro-CT examination confirmed reticular structure and monotonous high density at the central coagulation necrosis preserving air content and surrounding consolidation, and organization lesion of the IPM lesion.

**Conclusion:**

Our investigations suggest that RHS might be understood as a kind of immune reconstitution syndrome and be the initial and prior status of air crescent sign.

**Virtual Slides:**

The virtual slide(s) for this article can be found here: http://www.diagnosticpathology.diagnomx.eu/vs/3480054198968132

## Background

Reversed halo sign (RHS) has been regarded as a sign on computed tomography (CT) image characterized by central ground-glass opacity (GGO) which is surrounded by a partial or complete rim of consolidation. Although this sign was initially described specifically for cryptogenic organizing pneumonia [1], it has also been associated with other diseases, including fungal infections [2,3]. However, only a few studies have conducted a detailed pathophysiological investigation of this phenomenon. Therefore, the present report describes detailed radiological and histopathological findings of a case of invasive pulmonary mucormycosis (IPM) presenting RHS, with comparison to a discrete nodule (DN) [4,5] caused by invasive pulmonary aspergillosis (IPA), and discusses the pathophysiological implications of this characteristic image.

## Case presentation

**Figure 1:**
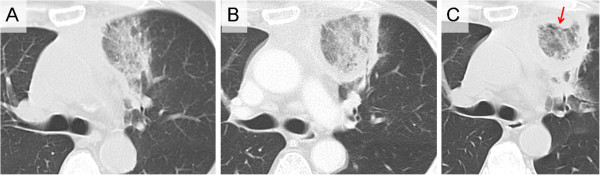
**Sequential chest computed tomography images in patient with invasive pulmonary mucormycosis presenting reversed halo sigh.** (**A**) On 39th (−39th) day before the operation, chest computed tomography (CT) revealed ground-glass opacity (GGO). (**B**) On -28th day, chest CT revealed reversed halo sign (RHS) comprising both central GGO and the outer rim recognized as a ring-shaped high-density area replacing the outside parenchyma of GGO. (**C**) On -11st day, chest CT revealed RHS air crescent-like small air slit (the tip of an arrow) at the junctional area between GGO and the outer rim.

**Figure 2:**
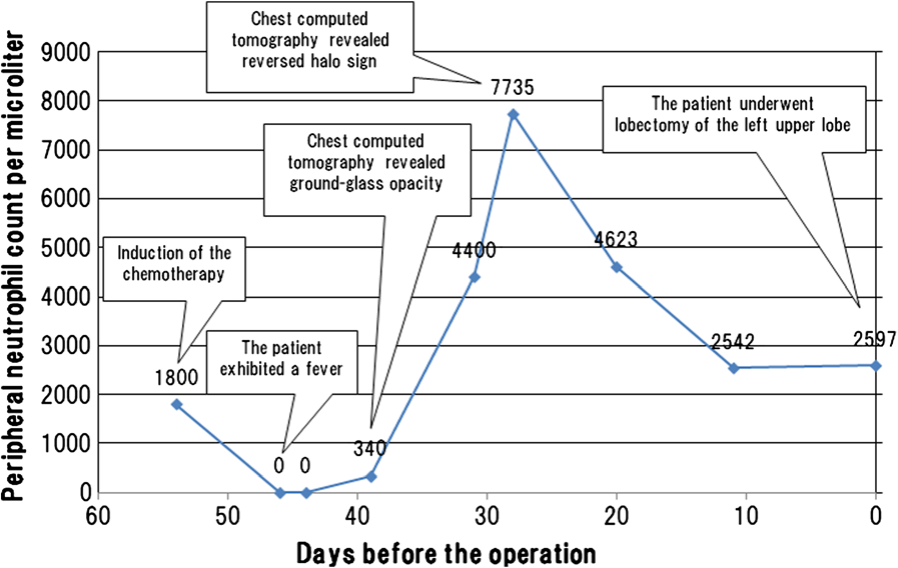
**A timeline visualizing the events presented in the case.** Chemotherapy was introduced on 54th day before the operation (−54th). On -46th day, the patient exhibited a fever (38°C), and chemotherapy-induced neutropenia was confirmed with 0 per microliter of his peripheral neutrophil count (PNC). On -39th day, chest computed tomography (CT) revealed ground-glass opacity in the left upper lobe. On -28th day, chest CT revealed reversed halo sign. Finally, the patient underwent the lobectomy of the left upper lobe. The PNC on the 54th, 46th, 39th, 28th day before the operation was 1800, 0, 340, and 7735 per microliter, respectively.

A 64-year-old Japanese man reported dyspnea and general fatigue two months before admission. He was referred to our hospital due to acute lymphoblastic leukemia, and chemotherapy was introduced in accordance with the CALGB 8811 protocol [6] on 54th day before the lobectomy of the left upper lobe (−54th). On -46th day, the patient exhibited a fever (38°C), and chemotherapy-induced neutropenia was confirmed with a peripheral neutrophil count (PNC) of 0 per microliter. Further, on -39th day, chest CT revealed GGO in the left upper lobe (Figure 1A). On -31st day, septum cytology revealed a small number of mold species and treatment comprising intravenous liposomal amphotericin B at 3 mg/kg/day was started. On -28th day, subsequent chest CT revealed RHS comprising both a central GGO and an outer rim recognized as a ring-shaped high-density area replacing the outside parenchyma of GGO (Figure 1B). The chest CT showed an increasing in thickness the outer rim on −20 day, and revealed RHS with air crescent-like small air slit between a part GGO and the outer rim on -11st day (Figure 1C), while serum Beta-D-Glucan levels failed to reach a normal level. The patient therefore underwent the operation. The PNC on the 54th, 46th, 44th, 39th, 31st, 28th, 20th, and 11st day before the operation was 1800, 0, 0, 340, 4400, 7735, 4623, and 2542 per microliter, respectively (Figure 2).

## Material and methods

### Tissue sample

Lung tissue from surgically removed the patient with IPM presenting RHS on a CT image was prepared for the present report. Additionally, lung tissue from autopsied patients with IPA was also employed to aid in a discussion of the pathophysiological implications of RHS. Namely, a representative sample of IPA was chosen among the lung tissues of 64 autopsies with IPA, which has been previously reported [4]. The selection was based on the following two criteria: one is a patient who had an agranulocytosis due to severe bone marrow suppression and another is the formalin-fixed tissue from IPA indicating a discrete nodular coagulation necrosis with surrounding of oozing; DN, which has been generally accepted as the lesion mirrored as CT halo sign [4,5]. In addition, written informed consent (in Japanese) was obtained from the patients’ family for publication of this study and any accompanying images. The case report was undertaken in compliance with the Helsinki Declaration.

### Histopathological examination

Routine histopathological examination was conducted. Briefly, the specimens were fixed with 10% formalin, embedded in paraffin wax after dehydration, and cut into 4 μm-thick sections. These were then prepared and stained with Hematoxylin and Eosin double, Elastica van Gieson, and Grocott stains for light microscopic observation.

### Image analyzing with micro-CT and 3D image reconstruction

The formalin-fixed tissues were dehydrated with 100% ethanol for 24 hours, dried for 3 hours at room temperature, and trimmed to a proper size. Scanning was carried out using a micro-CT scanner (InspeXio SMX-100CT, Shimadzu, Kyoto, Japan) with 45 kV tube voltage and 40 μA tube electric current. In the present study, the field of view was fixed at approximately six mm (XY plane) X five mm (Z plane) and the reconstruction matrix size was determined 512 × 512 pixels. After obtaining more than 400 two-dimensional (2 D) images of 12-μm thickness, a series of individual 2 D images were provided to a virtual three-dimensional (3 D) image [7]. Namely, parallel 2 D images were stacked using a commercially available 3 D reconstruction program (VG studio software, Nihon Visual Science, Tokyo, Japan) to generate 3 D images of the tissue samples. In the present report, since representative tissue samples from patients with IPM or IPA was prepared one from each lung tissue, a statistical analysis was not used.

## Results

**Figure 3:**
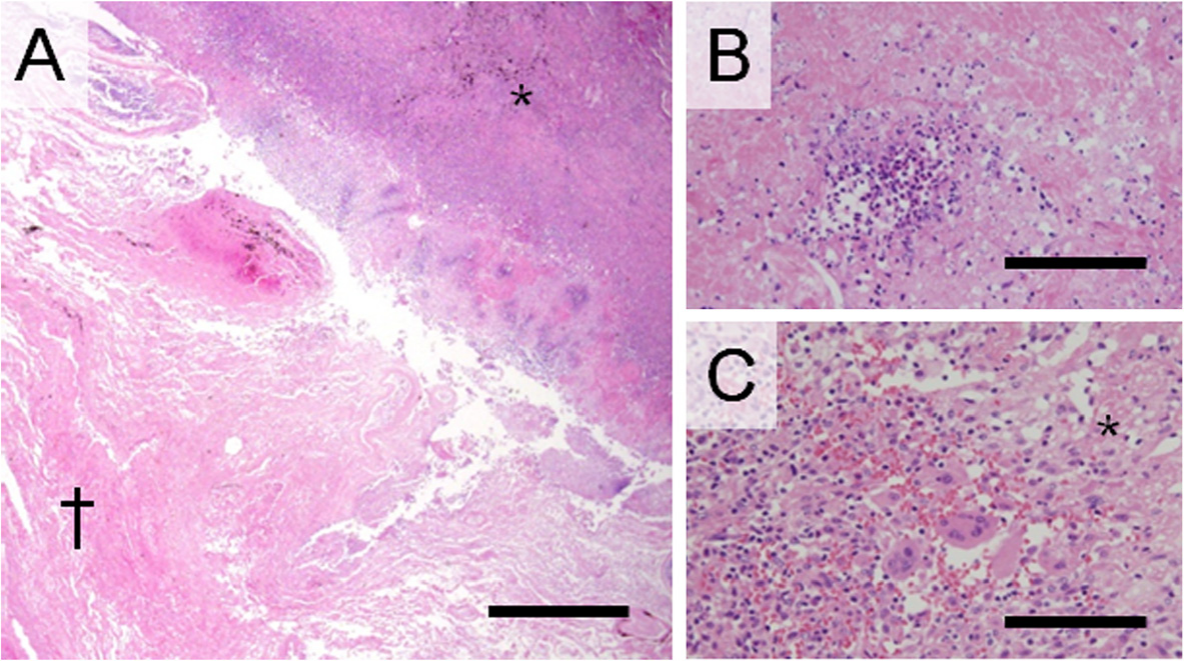
**Histopathological examinations in patient with invasive pulmonary mucormycosis presenting reversed halo sigh.** (**A**) The dagger (†) and the asterisk signs (*) are placed on the areas of ground-glass opacity (GGO) and outer rim on computed tomography image, respectively. The area of GGO comprised coagulation necrosis with remaining alveolar septa and air content. The outer rim comprised triplet structure; liquefaction, consolidation, and organization from the inner to the outer layer (Photomicrographs stained with hematoxylin and eosin (HE) double stain and scale bar represents 1000 μm). (**B**) High-power field of the consolidation, the second layer of the outer rim was constituent with mononuclear cell and multinucleated giant cell filling alveolar space (Photomicrographs stained with HE double stain and scale bar represents 100 μm). (**C**) High-power field of the liquefaction layer of the outer rim. Necrosis and neutrophil infiltration was observed, whereas no elastic fiber was observed (Photomicrographs stained with HE double stain and scale bar represents 100 μm).

**Figure 4:**
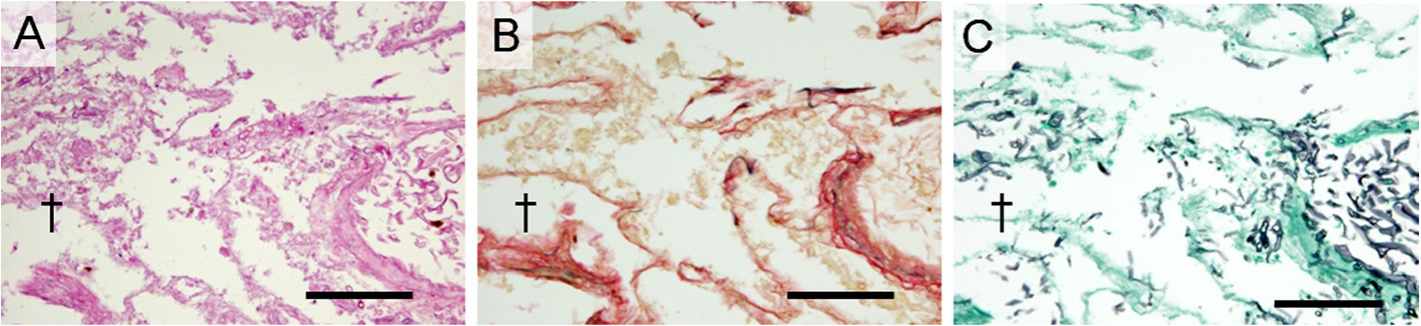
**Histopathological examinations of central coagulation necrosis area in patient with invasive pulmonary mucormycosis.** (**A**, **B**, and **C**) Histological examination of the surgical specimen presenting reversed halo sigh revealed pulmonary mucormycosis showing irregular, broad-based, and non septated hyphae with branching. Massive angioinvasion was observed in the coagulation necrosis background and alveolar septa and air content was remained. The dagger (†) sign is placed on the areas of ground-glass opacity on computed tomography image. (Photomicrographs stained with hematoxylin and eosin double, Elastica van Gieson, and Grocott stains, respectively. Scale bars represent 100 μm).

**Figure 5:**
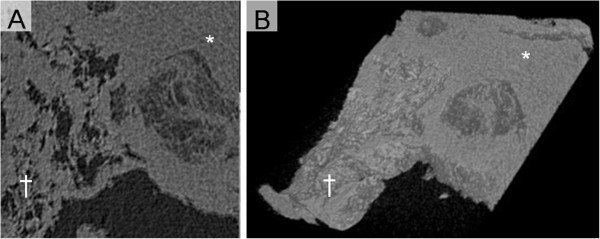
**Radiological examinations in patient with invasive pulmonary mucormycosis presenting reversed halo sigh.** (**A**) Micro computed tomography (CT) image of the lung tissue with invasive pulmonary mucormycosis. The dagger (†) and the asterisk signs (*) are placed on the areas of ground-glass opacity (GGO) and outer rim, respectively. (**B**) 3D reconstruction image processed by applying 3D volume rendering software. Central GGO and peripheral ring-shaped high-density area (*) were observed.

Histological examination of the pulmonary lesion that presented as CT-RHS, comprised coagulation necrosis of the alveolar septa and had remaining air content in the central area where had mirrored GGO (Figures 3A and 4). Periphery of the lesion corresponding to the outer rim comprised triplet structure; liquefaction, consolidation, and organization from the inner to the outer layer. There was no elastin in the liquefaction where developed at the junctional area between the coagulation necrosis and the consolidation (Figure 3B). The consolidation, the second layer of the outer rim was constituent with mononuclear cell and multinucleated giant cell filling alveolar space, but few were seen polymorphonuclear leukocytes (Figure 3C). In this layer, although elastic fibers were confirmed in each septum, they were altered in more or less. At the third layer of the outer rim, the alveolar space was replaced with collagen fibers with well maintaining of elastic fibers, which were major skeleton of alveolar septa. There were no inflammatory infiltrate in this layer. Micro-CT and that 3D reconstruction image confirmed both central GGO and peripheral ring-shaped high-density area of the IPM lesion, respectively (Figure 5).

**Figure 6:**
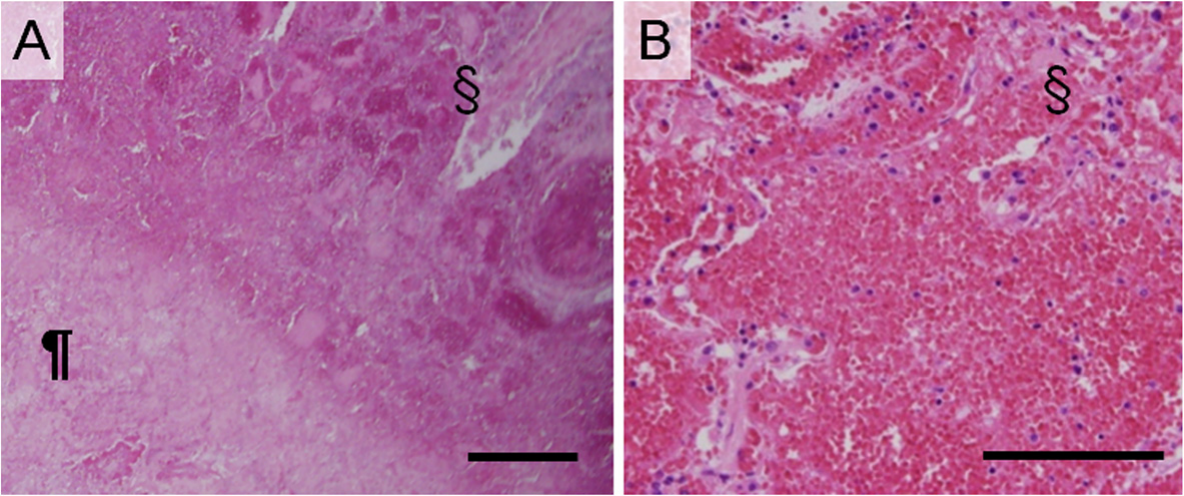
**Histopathological examinations in patient with invasive pulmonary aspergillosis presenting discrete nodule.** (**A**) The pilcrow (¶) and the section signs (§) are placed on the areas of monotonous high density and peripheral reticular structure on computed tomography image, respectively. The area of monotonous high density nodule (¶) comprised coagulation necrosis with remaining alveolar septa and without air content due to inflammatory exudation. The necrosis was surrounded by alveoli filled with oozing (§) (Photomicrographs stained with hematoxylin and eosin (HE) double stain and scale bar represents 1000 μm). (**B**) High-power field of peripheral oozing of red blood cell (Photomicrographs stained with HE double stain and scale bar represents 100 μm).

**Figure 7:**
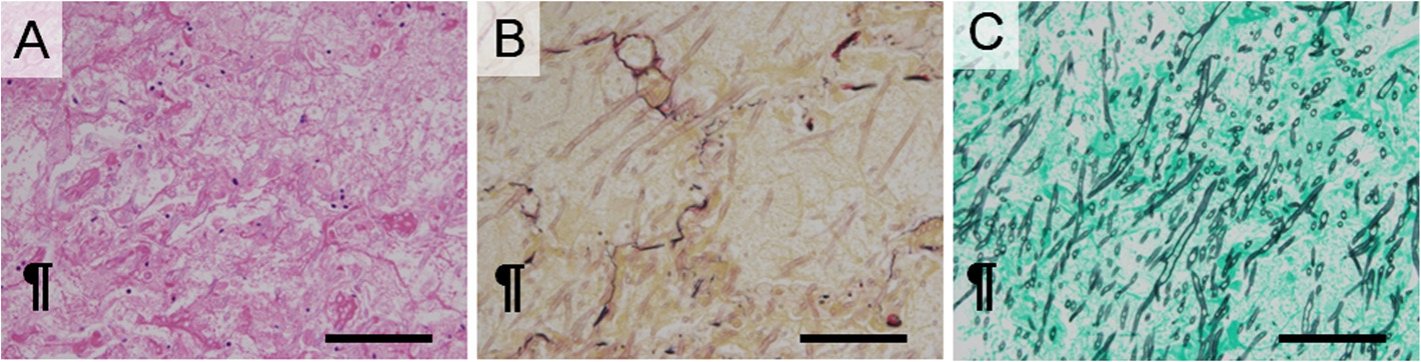
**Histopathological examinations of central coagulation necrosis area in patient with invasive pulmonary aspergillosis.** (**A**, **B**, and **C**) Histological examination of the lung tissue from autopsied patients with invasive pulmonary aspergillosis revealed pulmonary aspergillosis showing Y-shape hyphae with septa. Massive angioinvasion was observed in the coagulation necrosis background. Since alveolar space was filled by plasma exudation, the air content was not remained (Photomicrographs stained with hematoxylin and eosin, Elastica van Gieson, and Grocott stains, respectively. Scale bars represent 1000 μm).

**Figure 8:**
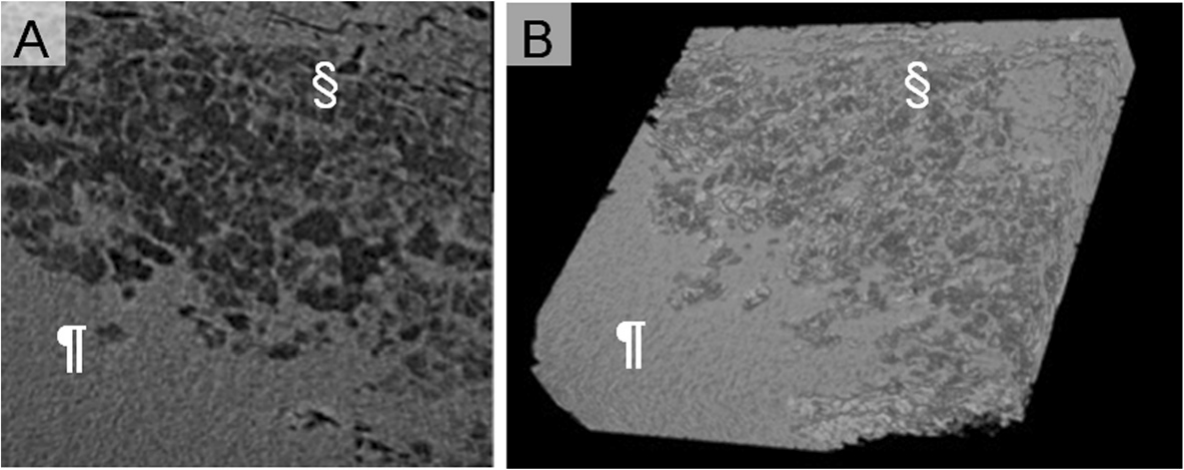
**Radiological examinations in patient with invasive pulmonary aspergillosis presenting discrete nodule.** (**A**) Micro computed tomography image of the lung tissue with invasive pulmonary aspergillosis. The pilcrow (¶) and the section signs (§) are placed on the areas of monotonous high density and peripheral reticular structure, respectively. (**B**) 3D reconstruction images processed by applying 3D volume rendering software. Central monotonous high density and peripheral fine reticular pattern were observed.

In contrast, although DN of IPA [5] also comprised central coagulation necrosis, air content in alveolar space was entirely replaced with exudation (Figure 6A), which likely comprised plasma without leukocytes (Figure 7). The round-shaped coagulation necrosis was encompassed with oozing of red blood cell (Figure 6B). Micro-CT and that 3D reconstruction image confirmed both monotonous high density and fine reticular pattern at center and periphery of the IPA lesion, respectively (Figure 8).

## Discussion

It has been accepted that RHS has appeared in CT images from immunocompromised patients indicates invasive fungal infection [3], including in patients with pulmonary mucormycosis [8]. In particular, Busca A et al. described that RHS may be an early radiological sign of mucormycosis and emphasized the importance of recognizing this sign as a diagnostic strategy [8]. However, detailed pathophysiology of the sign remains obscure. Therefore, we would like to discuss how RHS develops in comparison with the histopathological findings and micro-CT images of pulmonary lesions presented IPM or IPA. For our patient, the lesion presented RHS histologically comprised central coagulation necrosis remaining air content and ring-shaped peripheral consolidation or fibrosis, which was also confirmed by micro-CT images and their 3D reconstruction. The central GGO had been clinically noted during neutropenia prior to out-ward development of the outer rim, which was coincidently confirmed at the time of recovering of PNC. This fact may suggest that the recovery of impaired bone marrow function derived neutrophils at the periphery of coagulation necrosis, which might be induced by intravascular growth of invaded *Mucor*. Neutrophil provision might induce thin ring-shaped liquefaction between coagulation necrosis and adjacent unaltered parenchyma because there were no elastic fibers in the liquefaction, which was digested by neutrophil function. The liquefaction, logically understood as abscess was followed by the out-ward development of mononuclear cell infiltrate replacing alveolar space which as observed as the second layer of the outer rim of CT-RHS. This finding is generally accepted as a protective response against necrosis, but this was not developed inside the ring-shaped liquefaction because coagulation necrosis had no blood flow [9]. Further protective response was noted as the third layer of the outer rim, which can be named as organization [10]. The change is histologically defined as fibrosis entirely filling the alveolar space and well-remaining elastin in septa. Accordingly, pathophysiology of CT-RHS might be understood as a kind of immune reconstitution syndrome. In the case, the sign might mirror the recovering of impaired bone marrow functioning. On the other hand, it has been reported that recovery of neutrophil function in patients with IPA result in liquefaction and this process was recognized prior to the development of a CT-air crescent sign [5]. In the present IPM case, the CT image on -11st day revealed air crescent-like small air slit between GGO and the outer rim, which might be reflected by the excretion of liquefaction layer. It suggests that RHS might be the initial and prior status of CT-air crescent sign.

The present study also shed some light on the difference in both histological and micro-CT images between IPM and IPA in patients with neutropenia. The lung tissue histologically presented DN comprised nodule of coagulation without air content, where mirrored the nodule of monotonous high density on the micro-CT image. Accordingly, a central monotonous high-density nodule and GGO may be understood as a characteristic sign of IPA and IPM in patient with neutropenia, respectively. The difference should be derived whether alveolar space is filled or not filled with exudation. However, cause of exudation filling alveolar space in case of IPA is unclear, although the difference in intensity of penetration can be suggested between *Aspergillus* and *Mucor*[5]. Namely, *Aspergillus* usually shows a strong intensity of penetration in the lung regardless of a difference in compliance and/or elasticity for each component. Such strong invasiveness of *Aspergillus* may easily induce plasma exudation. In contrast, *Mucor* could penetrate though septal wall from lumen of capillary or small vascular, if exudation were seen in alveoli like IPA.

Meanwhile, we wish to go into insight of the usefulness of the molecular supplemental procedures on formalin-fixed and paraffin-embedded (FFPE) tissue sections. Whereas it has been largely accepted that microscopic findings of the hyphae (diameter, presence of septa, branching angle, and pigmentation) are important to determine causative agents in foci of invasive mold infections, it might be difficult to identify causative molds only by histopathological observation even that was made with the best effort. Histological identification might have a limitation itself [11]. Therefore, the establishment of an auxiliary diagnostic method for use in routine pathological laboratories is required. Previous investigators emphasized the usefulness of molecular biology methods on FFPE tissue sections [11-13]. Since FFPE sections are widely employed in routine preparations for surgical pathology, molecular biology methods may be utilized in many medical institutes. Furthermore, an additional diagnostic procedure will not be required if the stored paraffin-embedded tissue could be utilized for considerable molecular methods. Although further careful investigations are required, an establishment of accurate auxiliary molecular biology method could surely contribute to improve an accuracy of conventional histopathological diagnosis of this seriously debilitating infectious disease.

## Conclusion

Our study can suggest that reversed halo sign might be considered as a form of immune reconstitution phenomena, especially induced by recovering of bone marrow functioning, and be the initial and prior lesion of the air crescent sign.

### Consent

Written informed consent was obtained from the patient for publication of this Case report and any accompanying images. A copy of the written consent is available for review by the Editor of this journal.

## Abbreviations

RHS: Reversed halo sign; CT: Computed tomography; GGO: Ground-glass opacity; IPM: Invasive pulmonary mucormycosis; DN: Discrete nodule; IPA: Invasive pulmonary aspergillosis; PNC: Peripheral neutrophil count; FFPE: Formalin-fixed and paraffin-embedded.

## Competing interests

Dr. Shibuya reports receiving research grants from Janssen Pharmaceutical K.K., Dainippon Sumitomo Pharma Co., Astellas Pharma Inc., Taiho Pharmaceutical Co., and POLA-Pharma Inc. Other authors declare that they have no Conflict of Interests.

## Authors’ contributions

YO and TI equally conceptualized the case report, integrated the data, and completed the manuscript as a major contributor. Namely, YO and TI contributed equally to this work; HI contributed to management of the patient and revised clinical description; FS performed operation and contributed to management of the patient; KA evaluated radiological examinations and revised manuscript; DS, TA, MS, NT, and MW carried out the histopathological evaluation and revised histopathological description; YH contributed to management of the patient as a chief doctor of Department of Chest Surgery; HN and TN carried out the histopathological evaluation; KS integrated the data, revised manuscript, and gave final approval to the manuscript as a corresponding author. Furthermore, all authors contributed towards the conceptualization, writing, reading, and approval of the final manuscript.
